# Stimulation of *Codonopsis pilosula* Polysaccharide on *Bifidobacterium* of Human Gut Bacteria In Vitro

**DOI:** 10.1155/2021/9524913

**Published:** 2021-03-29

**Authors:** Jiankuan Li, Lina Dong, Yue Liu, Jianping Gao

**Affiliations:** ^1^School of Pharmaceutical Science, Shanxi Medical University, Taiyuan 030001, China; ^2^Central Laboratory, Shanxi Provincial People's Hospital, Affiliate of Shanxi Medical University, Taiyuan 030012, China; ^3^China Institutes of Biomedical Sciences, Shanxi University, Taiyuan 030006, China

## Abstract

**Objective:**

To evaluate the prebiotic effects of *Codonopsis pilosula* polysaccharide (CPP) on human gut bacteria in vitro.

**Methods:**

Codonopsis Radix was extracted with water at 100°C, and the extract was precipitated by 80% ethanol to obtain CPP. Human fresh fecal samples were collected from three healthy adults and used to ferment CPP. The fermented samples were collected to be analyzed by 16S rRNA sequencing.

**Results:**

The results showed that CPP exhibited significantly the stimulation on the growth of genus *Bifidobacterium* of human gut bacteria (*Padj* < 0.05). Although CPP also exhibited regulative trends on the genera including *Acidaminococcus*, *Bilophila*, *Dorea*, and *Eggerthella*, no significant differences were observed (*Padj* > 0.05), which was likely associated with the limited samples (*n* = 3).

**Conclusion:**

CPP has the potential to stimulate the growth of *Bifidobacterium* of the human gut bacteria and to be benefit to human health.

## 1. Introduction

Codonopsis Radix (commonly named Dangshen in Chinese), derived from the roots of *Codonopsis pilosula* (Franch.) Nannf., *Codonopsis pilosula* (Franch.) Nannf. Var. modesta (Nannf.) L. T. Shen, and *Codonopsis tangshen* Oliv., has been usually used in many traditional Chinese medicine preparations for tonifying the spleen and lung in China [1]. Polysaccharides are traditionally considered to be significant active components responsible for the tonifying function of Codonopsis Radix. Recently, increasing evidences indicated that *Codonopsis pilosula* polysaccharides exhibited a variety of biological activities. Zhang et al. reported that *Codonopsis pilosula* polysaccharides attenuated tau hyperphosphorylation and cognitive impairments in adeno-associated virus serotype-induced expression of human full length tau in C57/BL6 mice [2]. Fu et al. reported that *Codonopsis pilosula* polysaccharides exhibited the protection against cyclophosphamide-induced immunosuppression in mice [3]. Pectin-type polysaccharides from *Codonopsis pilosula* were reported to exhibit immune-stimulation activity in RAW264.7 macrophage and the selenizing *Codonopsis pilosula* polysaccharides protected RAW264.7 cells from hydrogen peroxide-induced injury [4, 5].

The human gastrointestinal tract is enriched with a complex population of microorganisms, which exert a significant influence on the host health and disease [6]. Previously, inulin-type fructans were obtained from *Codonopsis pilosula* and showed prebiotic activity by stimulating the growth of *Lactobacillus* and *Bifidobacterium* in vitro [7, 8]. Jing et al. reported that *Codonopsis pilosula* polysaccharides exhibited prebiotic-like effects in dextran sulfate sodium-induced colitis mice by stimulating the growth of *Bifidobacterium*, *Lactobacillus*, and *Akkermansia* probiotics [9]. The present study intends to investigate the modulation of CPP on human gut bacteria by in vitro fermentation.

## 2. Methods

### 2.1. Plant Material

Roots of *Codonopsis pilosula* (Franch.) Nannf. were collected from Pingshun county in Shanxi province, China, and were identified by Professor Jianping Gao (School of Pharmaceutical Science, Shanxi Medical University) to be Codonopsis Radix. A voucher specimen (2018-09-MCM) was deposited at Herbarium, School of Pharmaceutical Science, Shanxi Medical University.

### 2.2. Extraction of Polysaccharide

Dried roots of *Codonopsis pilosula* were crushed and extracted two times with water (10 : 1, v/w) at 100°C for 2 h. The combined extract was concentrated to 1/4 volume under vacuum at 60°C and then was precipitated with 80% ethanol and centrifuged for 15 min at 4000 rpm/min to obtain CPP. Proteins were removed from CPP using the Sevag reagent (chloroform/butanol, *v*/*v* = 4 : 1).

### 2.3. Fecal Sample Collection and Fermentation

Fresh fecal samples (10 g) were collected from three healthy adults and promptly suspended in pre-reduced phosphate-buffered saline (PBS). The samples were then added in 50 mL centrifuge tube pre-placed sterile filter bag and filtered. CPP was added in 100 mL filtered liquid at a ratio of 1 : 100 (g/mL) and fermented in anaerobic condition for 0, 8, and 24 h. After fermentation, 50 mL fermented liquid was filtered and the precipitate was added with 2 mL pre-reduced glycerin-PBS (1 : 1) and then centrifuged for 10 min at 4,000 rpm/min; the precipitate immediately was kept at −80°C for 16S rRNA gene sequencing analysis (DeepBiomedical Co., Ltd, Jinan, China).

### 2.4. 16S rRNA Sequencing

The DNA was extracted from 100 mg samples using DNeasy Power Soil Kit (Cat. 47014, Qiagen, Germany). The DNA concentration was measured with Nanodrop (Thermo scientific). The DNA was diluted to proper concentration for further 16S rRNA gene fragments (V3–V4) amplification. The polymerase used for 16S rRNA gene amplification was Phanta Max Master Mix (Vazyme Biotech Co., Ltd. Nanjing, China) following the manufacturer's procedure. The sequencing was implemented at Novogene Co. (Beijing, China) with a 2 × 250-bp paired-end sequencing strategy.

The V3-V4 regions of microbial 16S rDNA genes were amplified with primers of 341F (5′-CCTAYGGGRBGCASCAG-3′) and 806R (5′GGACTACNNGGGTATCTAAT-3′). The 25 *µ*l PCR amplification mixture contained 25 ng DNA, 1 *µ*l each primer (10 *µ*M), 0.5 *µ*l dNTP (2.5 mM), 12.5 *µ*l Vazyme Phata max buffer, and 0.5 *µ*l vazyme polymerase (Vazyme Biotech). The PCR was performed with an initial denaturation (5 minutes at 95°C), followed by 27 cycles of 15 seconds at 95°C, 15 seconds at 55°C, and 30 seconds at 72°C, and final with one cycle of 5 min at 72°C. The PCR products were purified with the KAPA Pure Beads (Roche) according to the manufacturer's instructions and further sequenced with an Illumina NovaSeq 6000 system (Illumina). The sequences were then clustered into OTUs (operational taxonomic units) at 99% identity thresholds using MOTHUR. Representative sequences of OTUs were selected and compared with ribosomal RNA database to obtain species annotation information. According to species annotation information, OTUs were filtered to obtained valid OTUs.

### 2.5. Statistical Analysis

Basing on the absolute abundance of valid OTUs and annotation information, the genus taxonomic level was obtained for differential abundance analysis with DEseq2 (version 1.26.0), *Padj* < 0.05 was considered to be significant difference.

## 3. Results and Discussion

### 3.1. Stimulation of CPP on the Growth of *Bifidobacterium* and *Acidaminococcus*

**Figure 1:**
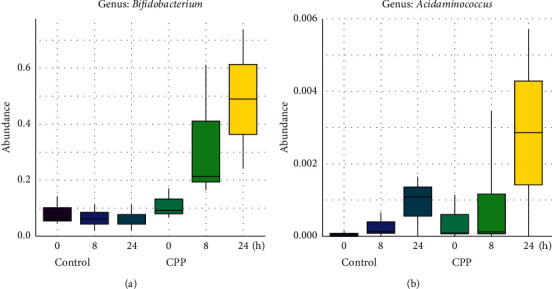
Stimulation of CPP on *Bifidobacterium* (a); control 24 versus CPP 24, *Padj* < 0.05) and *Acidaminococcus* (b).

As shown in Figure 1, the genus *Bifidobacterium* growth (Figure 1(a)) was observed to be significantly stimulated by CPP after fermentation for 8 and 24 h. The previous study by Jing et al. showed that *Codonopsis pilosula* polysaccharides exhibited the stimulation on the growth of *Bifidobacterium*, *Lactobacillus*, and *Akkermansia* probiotics in dextran sulfate sodium-induced colitis mice [9]. The present study showed that only *Bifidobacterium* of human gut bacteria was stimulated by CPP during 24 h fermentation. It was well known that *Bifidobacterium* was important probiotics. So, it was indicated that CPP was potential prebiotics by stimulating the growth of *Bifidobacterium* both in vivo and in vitro, which could be explained by the fact that Codonopsis Radix was rich in insulin-type fructans [8].

Meanwhile, CCP also exhibited a stimulatory trend on the growth of *Acidaminococcus* (Figure 1(b)) but no significant difference was observed (*Padj* > 0.05). *Acidaminococcus* was first reported by Rogosa to be using amino acid as the sole energy source for growth [10]. The effects of *Acidaminococcus* on human health were unclear. Previously, Qi et al. found that the composition of *Haemophilus*, *Lachnospira*, *Dialister*, and *Acidaminococcus* was decreased significantly in T1DM Chinese children compared to controls [11]. Li et al. found that the relative abundances of *Acidaminococcus*, *Succinivibrio*, and *Citrobacter* were reduced obviously in canines with high fat diet-induced obesity, while green tea polyphenols could increase the abundances of *Acidaminococcus*, *Succinivibrio*, and *Citrobacter* [12]. These studies indicated in some ways that *Acidaminococcus* could be associated negatively with metabolic diseases such as obesity and diabetes mellitus.

### 3.2. Inhibition of CPP on the Growth of *Bilophila*, *Dorea*, and *Eggerthella*

**Figure 2:**
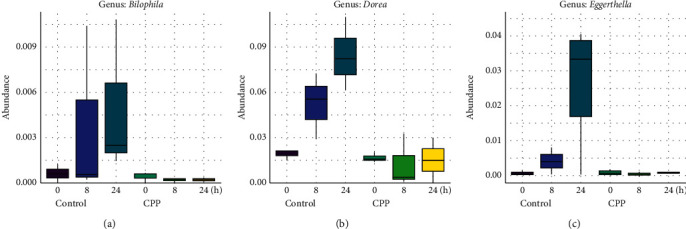
Inhibition of CPP on *Bilophila* (a)*, Dorea*, (b) and *Eggerthella* (c).

As shown in Figure 2, CPP exhibited inhibitory trends on the growth of three genera including *Bilophila* (Figure 2(a)), *Dorea* (Figure 2(b)), and *Eggerthella* (Figure 2(c)) after fermentation for 8 and 24 h, but no significant differences were observed (*Padj* > 0.05). In the control group, *Bilophila, Dorea* and *Eggerthella* exhibited a natural growth tendency, while they exhibited almost no growth tendency in CPP group. Natividad et al. reported that *Bilophila wadsworthia* with high-fat diet promoted higher inflammation, intestinal barrier dysfunction, and bile acid dysmetabolism, leading to higher glucose dysmetabolism and hepatic steatosis in mice [13], while the probiotic *Lactobacillus rhamnosus* CNCM I-3690 reduced *Bilophila wadsworthia*-induced immune and metabolic impairment. *Bilophila wadsworthia* was also reported to be more abundant in the colonic microbiome of colorectal cancer cases compared to healthy controls [14]. These data indicated that the genus *Bilophila* could likely be associated positively with inflammation.

Fang et al. compared the intestinal microbial diversity of healthy people and amyotrophic lateral sclerosis patients, which showed that the genus *Dorea* (harmful microorganisms) was significantly increased in amyotrophic lateral sclerosis patients [15]. The study by Chiu et al. showed that genus *Dorea* exhibited a significant correlation with allergic rhinitis [16]. Nitsan et al. found that the luminal proportions of *Faecalibacterium* and *Dorea* were significantly higher in fecal samples of irritable bowel syndrome (IBS) patient than those in fecal samples of healthy controls [17].

Sato et al. reported that the higher proportions of genera *Parabacteroides* and *Eggerthella* were associated with small newborn head circumference or weight in males [18]. *Eggerthella lenta*, a major cause of bacteremia, was reported to be a significant human pathogen that is often associated with serious gastrointestinal tract (GIT) pathology [19, 20].

CPP exhibited regulatory trends on the genera including *Acidaminococcus*, *Bilophila*, *Dorea*, and *Eggerthella*, but no significant differences were observed, which was likely associated with the limited samples in the study. So, it was suggested to increase samples in future studies to further investigate the modulation of CPP on the genera *Acidaminococcus*, *Bilophila*, *Dorea*, and *Eggerthella*.

## 4. Conclusion

CPP exhibits the potential to stimulate the growth of *Bifidobacterium* of the human gut bacteria and likely to be benefit to human health.

## Data Availability

The data used to support the findings of this study are available from the corresponding author upon request.
